# Implant–Abutment Interface Gels in Two-Piece Implant Systems: The “Guardian–Hazard” Paradigm—A Scoping Review

**DOI:** 10.3390/dj14100671

**Published:** 2026-10-14

**Authors:** Konstantinos Anastasiadis, Maria Kokoti, Athina Bakopoulou, Georgios Romanos

**Affiliations:** 1 Private Dental Practice, 15354 Glyka Nera, Greece; 2 Department of Prosthodontics, School of Dentistry, Faculty of Health Sciences, Aristotle University of Thessaloniki, 54124 Thessaloniki, Greece; mkokoti@dent.auth.gr; 3 Department of Periodontics and Endodontics, School of Dental Medicine, Stony Brook University, 106 Rockland Hall, Stony Brook, NY 11794, USA; georgios.romanos@stonybrookmedicine.edu

**Keywords:** chlorhexidine, dental implants, dental implant–abutment design, dental leakage, silicone gels, tribocorrosion

## Abstract

**Background/Objectives:** Antiseptic gels applied at the implant–abutment interface (IAI) aim to reduce microbial leakage and peri-implant inflammation. However, introducing viscous media into a precision screw joint may affect tribology, preload maintenance, mechanical stability and corrosion-related processes. This scoping review mapped evidence on chlorhexidine (CHX) and other IAI gels, considering their biological, mechanical and electrochemical effects. **Methods:** Following PRISMA-ScR and a Population–Concept–Context framework, in vitro, in vivo and clinical studies of two-piece implant systems were eligible if they evaluated CHX or another sealing gel at the IAI and reported microbial, mechanical or electrochemical outcomes. English-language full texts published between 2015 and 2025 were identified through PubMed/MEDLINE, Scopus, ScienceDirect and reference screening. Findings were synthesised qualitatively within “Guardian” (biological protection) and “Hazard” (mechanical or electrochemical effects) domains. **Results:** Of 241 records, 16 met the inclusion criteria. CHX gels reduced intra-implant bacterial counts, although their sealing effect was limited and time-dependent. Silicone-based sealants generally achieved better microbial leakage control and, in several studies, maintained or increased reverse torque values compared with dry controls. Conversely, aqueous or oil-based antiseptic agents frequently reduced reverse torque, suggesting lubrication-related effects on screw-joint stability. No study directly quantified tribocorrosion associated with specific gels. **Conclusions:** IAI gels may provide biological protection, but their effects depend on formulation and are not exclusively antimicrobial. Silicone-based materials demonstrated the most favourable combined microbial and mechanical performance. Tribocorrosion remains insufficiently investigated, highlighting the need for integrated microbial, mechanical and electrochemical studies.

## 1. Introduction

The rehabilitation of edentulous spaces with osseointegrated dental implants has become a predictable and highly successful treatment modality in modern dentistry. However, despite high survival rates, the long-term success of implant-supported restorations is frequently compromised by both biological and technical complications [1,2,3]. Long-term observations indicate that although biological integration is usually achieved, mechanical failures and peri-implant inflammation remain persistent challenges beyond five years of function [1,2].

Central to these complications is the architectural nature of the two-piece implant system [4,5]. The junction between the fixture and the prosthetic abutment—the Implant–Abutment Interface (IAI)—is generally not hermetically sealed [4,6]. Regardless of the connection design, whether external hex or internal conical, a microscopic gap has been consistently demonstrated at the interface of two-piece implant systems [5,6]. Clinical split-mouth data further suggest that implant–abutment connection geometry and platform morphology may influence crestal bone-level changes [7]. This microgap serves as a bidirectional pathway. Fluids and bacteria can penetrate the internal cavity of the implant, establishing an anaerobic reservoir [4,8,9,10]; while during functional loading, toxins and bacterial products may subsequently be expelled into the surrounding peri-implant tissues. This phenomenon, often described as the “pumping effect”, has been proposed as an important contributing etiological factor in crestal bone resorption and peri-implant inflammation [5,8,9,10].

To mitigate these biological risks, various protocols have been proposed and are widely used in clinical practice, including the application of antimicrobial agents to the IAI prior to abutment torquing [11,12,13]. Chlorhexidine (CHX) gels and varnishes are among the most commonly used agents, due to their broad-spectrum antimicrobial activity [11,12,13,14,15,16,17,18]. The clinical rationale is that by filling the microgap with an antiseptic medium, one aims to markedly reduce the initial bacterial load and delay recolonization, thereby acting as a “Microbial Guardian” for the peri-implant hard and soft tissues [11,12,13,19].

However, the introduction of a viscous medium into a complex mechanical joint introduces an additional mechanical variable that is often underrepresented in biologically oriented studies: tribology, the science of friction, lubrication, and wear between contacting surfaces [20,21,22,23]. The stability of the implant–abutment assembly relies heavily on the preload generated within the abutment screw, i.e., a tensile force produced by the applied tightening torque [20,24]. Fundamental engineering principles dictate that the presence of a lubricant between mating metal surfaces significantly alters the coefficient of friction [22,23,25]. While lubrication can theoretically increase preload for a given torque [22,26,27,28], it may, under certain conditions, also reduce the frictional resistance to screw rotation during dynamic cyclic loading, potentially facilitating screw loosening [20,21,22,23,25,26].

Thus, a critical clinical dilemma emerges. By applying a gel to serve as a biological seal, clinicians may inadvertently be introducing a potential “Mechanical Hazard,” as suggested by in vitro studies on CHX-containing gels, predisposing the restoration to screw loosening, a complication that affects a significant percentage of single-implant restorations [3,29,30,31].

Furthermore, this mechanical instability does not occur in isolation but precipitates a secondary, equally detrimental phenomenon: tribocorrosion. Cyclic micromotion at the IAI can disrupt the protective titanium oxide (TiO_2_) passivation layer, exposing the underlying bulk metal to the corrosive oral environment. The synergistic interaction of wear and corrosion may release cytotoxic titanium particles and ions into the peri-implant tissues, a process known to drive inflammation and bone loss [32]. Crucially, the introduction of acidic or fluoride-containing antiseptic agents may accelerate this degradation by chemically attacking the passivation layer, potentially compromising the stability of the passivation layer and accelerating electrochemical degradation processes [33].

Despite the clinical use of interface gels, the available evidence remains heterogeneous and fragmented. Most available studies investigate these phenomena in isolation rather than in an integrated framework: microbiological or microleakage assays focus primarily on leakage prevention and surrogate inflammatory outcomes, whereas mechanical and tribological studies concentrate on torque and reverse-torque values with little integration of biological endpoints [4,11,24,34,35,36,37]. Consequently, current evidence does not support a clear consensus regarding the overall safety and efficacy of interface gels [34,35,38,39,40,41].

This review aims to bridge this interdisciplinary gap. By critically synthesising data from recent in vitro, in vivo, and clinical studies, this review addresses the following question: whether CHX and related interface gels primarily function as antimicrobial “guardians” of peri-implant tissues or whether they may act as mechanical “hazards” that compromise the stability of the implant–abutment assembly.

## 2. Materials and Methods 

Study design and reporting framework: This scoping review was conducted and reported in accordance with the Preferred Reporting Items for Systematic Reviews and Meta-Analyses Extension for Scoping Reviews (PRISMA-ScR) [42]. The completed PRISMA-ScR checklist is provided as Supplementary Materials S2. The review protocol was not prospectively registered. The research strategy was structured according to a PCC (Population, Concept, Context) framework, as summarised in Table 1. The review addressed three guiding questions: (i) whether antiseptic gels effectively prevent microbial leakage at the implant–abutment interface; (ii) whether these agents affect the mechanical stability of the implant–abutment assembly; and (iii) whether antiseptic gels or related agents influence corrosion or electrochemical behaviour at the implant–abutment interface.

Eligibility criteria: Studies were considered eligible if they met all of the following inclusion criteria: (i) articles published between 1 January 2015 and 12 December 2025; (ii) articles published or available in English; (iii) primary research articles (in vitro, in vivo, or clinical trials); (iv) clearly identified experimental agents, focusing on CHX gels, silicone sealants, or similar antimicrobial interface agents; (v) assessment of at least one relevant outcome, including microbial sealing ability (microleakage), mechanical properties (e.g., reverse torque values or screw loosening), or electrochemical behaviour (e.g., fretting corrosion or titanium release); (vi) inclusion of an appropriate control group (e.g., dry connection or saliva/blood contamination without gel); and (vii) availability of full text.

Exclusion criteria: Studies were excluded if they met any of the following criteria: (i) publication before 1 January 2015; (ii) lack of full-text availability; (iii) languages other than English; (iv) reviews, systematic reviews, book chapters, conference papers, letters, notes or short communications; (v) studies in which the experimental gel formulation was unclear or non-commercial (unless the explicit aim was compound analysis); (vi) studies dealing exclusively with cement-retained restorations where the implant–abutment interface was not the primary variable; or (vii) absence of a control group.

Information sources and search strategy: Electronic searches were conducted in PubMed/MEDLINE and Scopus, supplemented by a search in ScienceDirect, for records published between 1 January 2015 and 12 December 2025; the searches were performed on 13–14 December 2025. PubMed/MEDLINE was selected to capture the biomedical and dental literature, whereas Scopus provided broader multidisciplinary coverage, including materials science and engineering. ScienceDirect was not treated as a primary bibliographic database but as a complementary full-text platform for identifying potentially relevant studies addressing tribological and corrosion-related aspects of implant systems. This combination was selected to cover the biomedical, dental, materials-science and engineering dimensions of the review question. Search strategies combined controlled vocabulary and free-text terms related to the implant–abutment interface, CHX gels, silicone-based sealants and antimicrobial interface agents. The search strings were tailored to each source and included Boolean operators to identify studies reporting microbial leakage, mechanical outcomes or electrochemical behaviour at the interface. In addition, the reference lists of all included articles and relevant reviews were screened to identify further eligible studies. The complete electronic search strategy for each source, including the exact search strings, fields searched, limits, search dates and yields, is provided in Supplementary Table S1.

**Figure 1:**
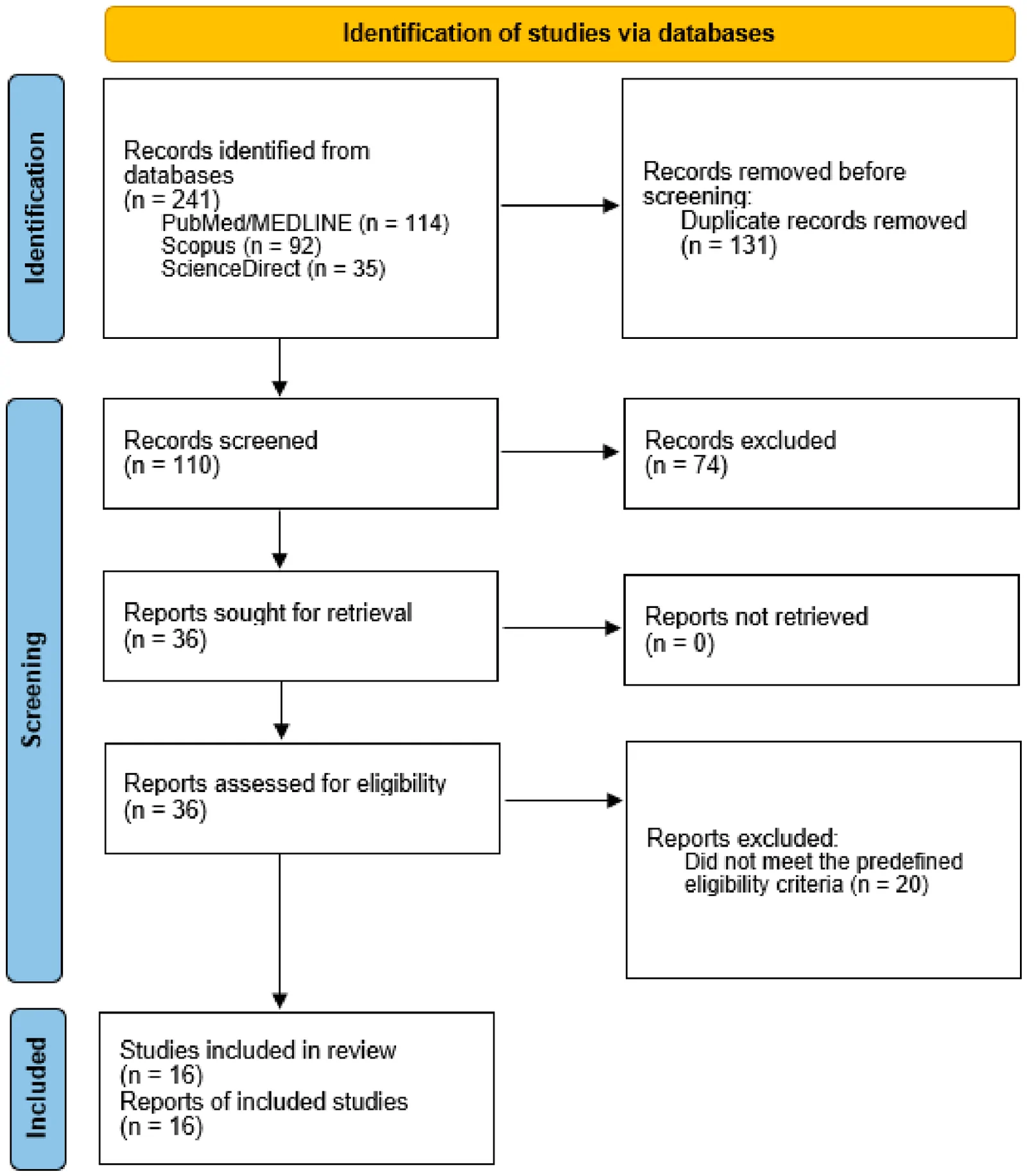
PRISMA-ScR flow diagram of study selection. Flow of records through identification, screening, eligibility assessment and inclusion. A total of 241 records were identified through database searches; after removal of 131 duplicates, 110 records remained for screening. Following title and abstract screening, 36 full-text articles were assessed for eligibility, and 16 studies met the predefined criteria and were included in the qualitative synthesis. Adapted from Page et al. [43] under the Creative Commons Attribution 4.0 licence.

Selection of sources of evidence: Titles and abstracts retrieved from the searches were screened for eligibility by a single reviewer. Potentially relevant articles were obtained in full text and re-assessed against the predefined inclusion and exclusion criteria. Where necessary, eligibility decisions were discussed among the authors. Reasons for exclusion at the full-text stage (e.g., inappropriate study design, absence of relevant outcomes, unclear gel formulation) were documented. The overall selection process is illustrated in a PRISMA-style flow diagram (Figure 1).

Data charting and synthesis: Data from the included studies were charted using a structured extraction approach. The following items were recorded where available: study design (in vitro, in vivo, clinical), implant system and connection type, type and commercial name of the interface agent, concentration, control condition(s), primary outcome domain (microbial, mechanical, electrochemical), secondary outcomes, and main conclusions. Studies were then categorised according to the predominant effect observed as “Guardian” (antimicrobial benefit), “Hazard” (mechanical and/or corrosion-related risk), or combined (Supplementary Materials, Table S2).

Given the heterogeneity of designs, outcomes and measurement protocols, no quantitative synthesis was attempted. Instead, findings were synthesised qualitatively, focusing on the comparative performance of CHX gels, silicone-based sealants and other agents with respect to microleakage prevention, torque maintenance and interface-related electrochemical phenomena.

No formal risk-of-bias assessment was undertaken, consistent with the exploratory aim of a scoping review; however, methodological features affecting interpretation (e.g., study design, sample size and loading protocols) were considered narratively.

Commercial product information: Information on commercial products was obtained from manufacturers’ product pages, instructions for use (IFU) and/or safety data sheets (SDS). For transparency, the source document(s) for each entry and the access date are provided in Supplementary Table S3.

## 3. Results

### 3.1. Overview of Included Evidence

Of the 16 included studies (Table 2), two investigated non-CHX sealing materials outside conventional silicone-based products: an in vitro study evaluating a siloxane/silica/paraffin varnish (Kiero Seal), which demonstrated reduced bacterial infiltration at a conical implant–abutment interface compared with unsealed controls, and an in vitro study examining a thermosensitive chitosan-based hydrogel (CS/β-GP/PVP–I), which showed antibacterial and sealing effects and increased removal torque values (RTVs) in selected formulations.

Fourteen studies were in vitro investigations: seven primarily evaluated microbial leakage, five focused on mechanical outcomes (removal torque, reverse torque values or screw loosening), and two assessed both microbial and mechanical endpoints within the same experimental design. Two clinical studies evaluated intra-implant bacterial contamination and/or peri-implant parameters in vivo.

CHX-based preparations were assessed in eight studies, whereas silicone-based sealants were investigated in at least six studies; several investigations evaluated more than one agent. Internal implant connections were more frequently represented than external connections, although implant–abutment designs and torque protocols varied substantially. Follow-up intervals and loading regimens were similarly heterogeneous, ranging from static assembly without loading to extended cyclic loading under simulated masticatory conditions. 

This heterogeneity supported the decision to undertake a qualitative rather than quantitative synthesis and informed the Guardian/Hazard categorisation adopted in this review.

### 3.2. Study Selection

The systematic article selection process is illustrated in Figure 1. Database searches in PubMed/MEDLINE, Scopus and ScienceDirect identified 241 records (PubMed *n* = 114; Scopus *n* = 92; ScienceDirect *n* = 35). After removing duplicates (*n* = 131), 110 records remained for title and abstract screening, of which 74 were excluded. Following the full-text evaluation of the remaining 36 articles, 16 studies met the inclusion criteria and were included in the qualitative synthesis.

Among the included articles, CHX gel was the most frequently investigated agent, typically evaluated through microleakage and torque-maintenance protocols. CHX concentrations ranged from 0.2% to 1% [11,44]. Silicone-based sealing agents represented the main alternative approach, and were frequently investigated in studies focusing on mechanical integrity or sealing capacity. These materials were evaluated either in comparison with CHX or as standalone agents intended to mitigate the microgap “pumping effect” [16,45]. 

A methodological distinction was observed between studies addressing the “Guardian” and “Hazard” domains. Studies focusing on antimicrobial protection primarily used microbial leakage models and, in clinical investigations, radiographic peri-implant parameters [6,40]. In contrast, studies examining potential mechanical hazards predominantly employed mechanical testing protocols, measuring RTV following dynamic cyclic loading [34,46].

### 3.3. Assessment of the “Guardian” Effect: Antimicrobial and Sealing Efficacy

The capacity of interface gels to act as “Microbial Guardians” was evaluated through both in vitro leakage models and in vivo clinical trials. Across the included studies, CHX gels demonstrated consistent antimicrobial activity but limited long-term sealing capacity compared with silicone-based sealants. 

In clinical settings, intra-operative application of CHX gel reduced bacterial contamination within the internal implant cavity and was associated with improvements in peri-implant inflammatory parameters in long-term follow-up [40]. However, some studies indicated that this antimicrobial effect may diminish over time, with endotoxin penetration eventually approaching levels observed in control groups [47]. 

In vitro studies similarly reported that CHX gels delay bacterial migration, but do not produce a hermetic seal at the interface. Comparative experiments showed that silicone-based agents (GapSeal) achieved superior microleakage control under certain test conditions, whereas CHX gels or varnishes eventually allowed bacterial penetration [16,48]. These findings suggest that the protective effect of CHX is primarily bactericidal, whereas silicone sealants offer a physical barrier. 

Emerging materials such as siloxane-based varnishes (Kiero Seal) [49] and thermosensitive chitosan hydrogels [50] have also demonstrated promising antibacterial and sealing performance at the IAI.

**Table 2:** Summary of included studies evaluating implant–abutment interface (IAI) gels (studies listed in chronological order).

First Author (Year)	Implant Connection	Type of Study	Study Focus	Gel/Material	Key Finding
Clinical/In Vivo Studies (*n* = 2)					
1. Ghannad et al. (2015) [11]	Conical	Clinical	Microbial	CHX Gel	Significant intra-operative reduction in bacterial counts in the internal cavity. (Guardian)
2. D’Addazio et al. (2023) [40]	Internal hex	Clinical	Microbial	CHX Gel	Clinical use of CHX gel showed reduction in peri-implant bacterial complications over 5 years. (Guardian)
In Vitro Studies (*n* = 14)					
3. Koutouzis et al. (2015) [47]	Conical	In vitro	Microbial	CHX Gel	CHX reduced endotoxin penetration but efficacy diminished over time compared to controls. (Mixed)
4. Podhorsky et al. (2016) [44]	Internal hex	In vitro	Microbial	CHX/Disinfectant	Disinfectant/sealant agents reduced bacterial transfer immediately after application. (Guardian)
5. Asli et al. (2017) [38]	Internal hex	In vitro	Mechanical	CHX Gel	CHX gel caused a significant reduction in detorque values, posing a screw loosening risk. (Hazard)
6. Antonio Ferreiraet al. (2017) [49]	Conical	In vitro	Microbial	Kiero Seal	Effective reduction in bacterial infiltration vs. unsealed control. (Guardian)
7. Mohammadi et al. (2019) [51]	Internal hex	In vitro	Microbial	GapSeal vs. CHX	GapSeal provided more durable sealing against bacterial leakage than CHX gel or Atridox. (Guardian)
8. Cao et al. (2019) [50]	Flat	In vitro	Microbial + Mechanical	Experimental Chitosan Hydrogels	One of the experimental hydrogels showed the best sealing and increased removal torque. (Guardian)
9. Seloto et al. (2020) [52]	External hex	In vitro	Mechanical	Sealing Agent	Evaluated vertical misfit; agents must be carefully applied to not interfere with passive fit. (Hazard)
10. Raju et al. (2021) [16]	Internal hex	In vitro	Microbial	Silicone vs. CHX	Silicone gel showed superior sealing ability compared to chlorhexidine varnish. (Guardian)
11. Ozdiler et al. (2021) [46]	Various conical	In vitro	Mechanical	Silicone vs. CHX	Silicone sealants maintained torque better than CHX in certain conical connections. (Mixed: connection-dependent)
12. D’Ercole et al. (2022) [6]	Various conical	In vitro	Microbial	Gel agents	Confirmed leakage patterns; gel application mitigated initial bacterial ingress in different connections. (Guardian)
13. Smojver et al. (2022) [48]	Conical & straight	In vitro	Microbial	GapSeal	Only GapSeal was found to significantly prevent microleakage compared to other agents (Flow.sil, Oxysafe). (Guardian)
14. Yu et al. (2022) [53]	Internal hex & conical	In vitro	Microbial + Mechanical	Silicone Gel	Sealing gel improved leakage resistance without compromising mechanical stability under dynamic loading. (Guardian)
15. Nourizadeh et al. (2022) [34]	Conical	In vitro	Mechanical	Oil/Water Agents	Application of antibacterial agents significantly reduced removal torque values compared to dry controls. (Hazard)
16. Katebi et al. (2024) [45]	Conical with internal octagon	In vitro	Mechanical	GapSeal	GapSeal application resulted in lower torque loss (higher RTV) compared to unsealed groups (Guardian).

### 3.4. Assessment of the “Hazard” Effect: Mechanical Stability

The potential for these agents to act as “Mechanical Hazards” was investigated primarily through the measurement of RTV. Several studies reported that the application of aqueous or oil-based antiseptic agents reduced RTV compared with dry control conditions. These findings were attributed to lubrication effects that decrease friction at the screw interface and may facilitate screw loosening during cyclic loading. Specifically, disinfection agents were reported to significantly reduce detorque values, thereby increasing the risk of screw loosening compared to non-lubricated interfaces [34,38]. Conversely, results for high-viscosity silicone sealants were generally more favourable. While reduced torque stability was observed in steep conical connections in some experiments [46], other recent studies demonstrated that GapSeal maintained RTV comparable to dry controls [45]. 

Additional in vitro investigations involving multiple implant systems demonstrated that certain sealing gels improved leakage resistance without compromising mechanical behavior under load [53]. In selected formulations, the thermosensitive chitosan hydrogels increased RTVs [50], suggesting that under some conditions a gel may contribute to mechanical stability rather than acting purely as a lubricant. No study within the included sample directly evaluated tribocorrosion or corrosion rates associated with the application of these specific interface gels, highlighting a significant gap in the available experimental evidence.

### 3.5. Commercial Landscape

Beyond conventional antiseptic agents such as CHX, several proprietary sealing materials have recently been introduced to address microbial colonisation at the implant–abutment interface. Silicone-based products are specifically marketed for their ability to seal the microgap and limit bacterial penetration. Available information regarding the composition and evidentiary status of these products is summarised in Table 3.

## 4. Discussion

The present review aimed to critically evaluate the dual role of implant–abutment interface (IAI) agents, balancing the antimicrobial benefits (“Guardians”) against potential biomechanical risks (“Hazards”). The synthesis of the included studies reveals a complex clinical reality: although the biological rationale for using interface gels is well established, their mechanical implications remain insufficiently investigated and may significantly influence the long-term stability of the implant–abutment assembly.

### 4.1. The “Guardian” Effect: Chemical vs. Physical Sealing 

The reviewed literature indicates that CHX gels function primarily as immediate chemical decontaminants rather than durable sealing agents. In vitro studies have demonstrated that the intra-operative application of 1% CHX gel significantly reduces the initial bacterial load within the implant cavity [11,44]. Long-term clinical observations further suggest that this early decontamination contributes to lower rates of peri-implant mucositis over a 5-year period [40]. However, in vitro findings indicate that the antimicrobial effect of aqueous CHX diminishes over time, likely due to gradual washout associated with the microgap “pumping effect” [47].

In contrast, silicone-based sealants appear to achieve a more stable “Guardian” effect through physical sealing. Comparative studies demonstrated that high-viscosity silicone matrices significantly delayed or prevented microbial leakage where aqueous gels were less effective [48,51]. Additionally, silicone agents outperformed CHX varnishes [16]. These findings suggest that long-term protection against bacterial recolonisation is influenced more strongly by material viscosity and hydrophobicity than by intrinsic antimicrobial chemistry alone.

### 4.2. The “Hazard” Effect: The Lubrication Dilemma

One of the most important findings of this review concerns the potential mechanical consequences of applying interface gels. The introduction of fluid or semi-fluid materials inevitably alters the tribology of the screw joint, particularly the coefficient of friction (K) during tightening. Evidence regarding aqueous and oil-based antiseptic agents, including CHX preparations, consistently points to a mechanical hazard. These agents act as lubricants, reducing RTVs relative to dry control conditions [34,38]. This confirms the concern that while lubrication might increase initial preload, it reduces the frictional resistance and may facilitate screw loosening under cyclic functional loading.

The mechanical behaviour of silicone-based sealants appears more complex. Several studies reported that GapSeal maintained or even improved RTVs compared with dry controls in internal connection systems [45,51]. In these cases, the highly viscous matrix may act as a damping medium, partially absorbing micromovements at the interface and contributing to preload stability.

Conversely, in certain steep conical connections, the application of silicone sealants has resulted in lower torque values than those observed with CHX-based gels [46]. One plausible explanation is that, within a tapered interface, incompressible silicone may behave as a hydraulic wedge when excessive material is interposed between the mating surfaces. This may hinder seating of the abutment, increase vertical misfit and reduce the effective metal–metal contact area [52]. Consequently, even small variations in sealant volume, distribution or taper geometry may influence torque retention. 

Overall, these observations suggest that the mechanical behaviour of silicone-based gels is highly dependent on implant connection design (conical versus flat-to-flat) and on the amount of material interposed at the interface. As such, their effects cannot be generalised across all implant–abutment configurations.

Connection design should not, however, be treated as a simple binary modifier. External-hex and other flat-to-flat interfaces rely predominantly on screw-generated preload and may be particularly susceptible to vertical misfit when an excessive volume of material is interposed between the mating surfaces [52]. Internal-hex designs provide greater geometric engagement, although the reviewed findings remained formulation-dependent: silicone-based sealants improved leakage resistance or maintained mechanical stability in several internal systems [16,51,53], whereas CHX-containing media reduced detorque values in others [38]. Conical connections derive part of their stability from frictional engagement between tapered surfaces; consequently, sealant viscosity, applied volume, taper angle and anti-rotational geometry may influence complete seating and torque retention. The divergent findings reported across conical systems [45,46] should therefore not be interpreted as a uniform effect applicable to all internal conical connections.

### 4.3. The Missing Link: Tribocorrosion

A notable gap identified in this review is limited direct evidence linking specific commercial interface gels to tribocorrosion processes. While the mechanisms of fretting corrosion at the interface are well documented [32], and simulated studies suggest that acidic environments or ionic debris may accelerate metal degradation [33], few investigations have directly evaluated the influence of commercial CHX or silicone-based gels within tribocorrosion testing systems.

**Figure 2:**
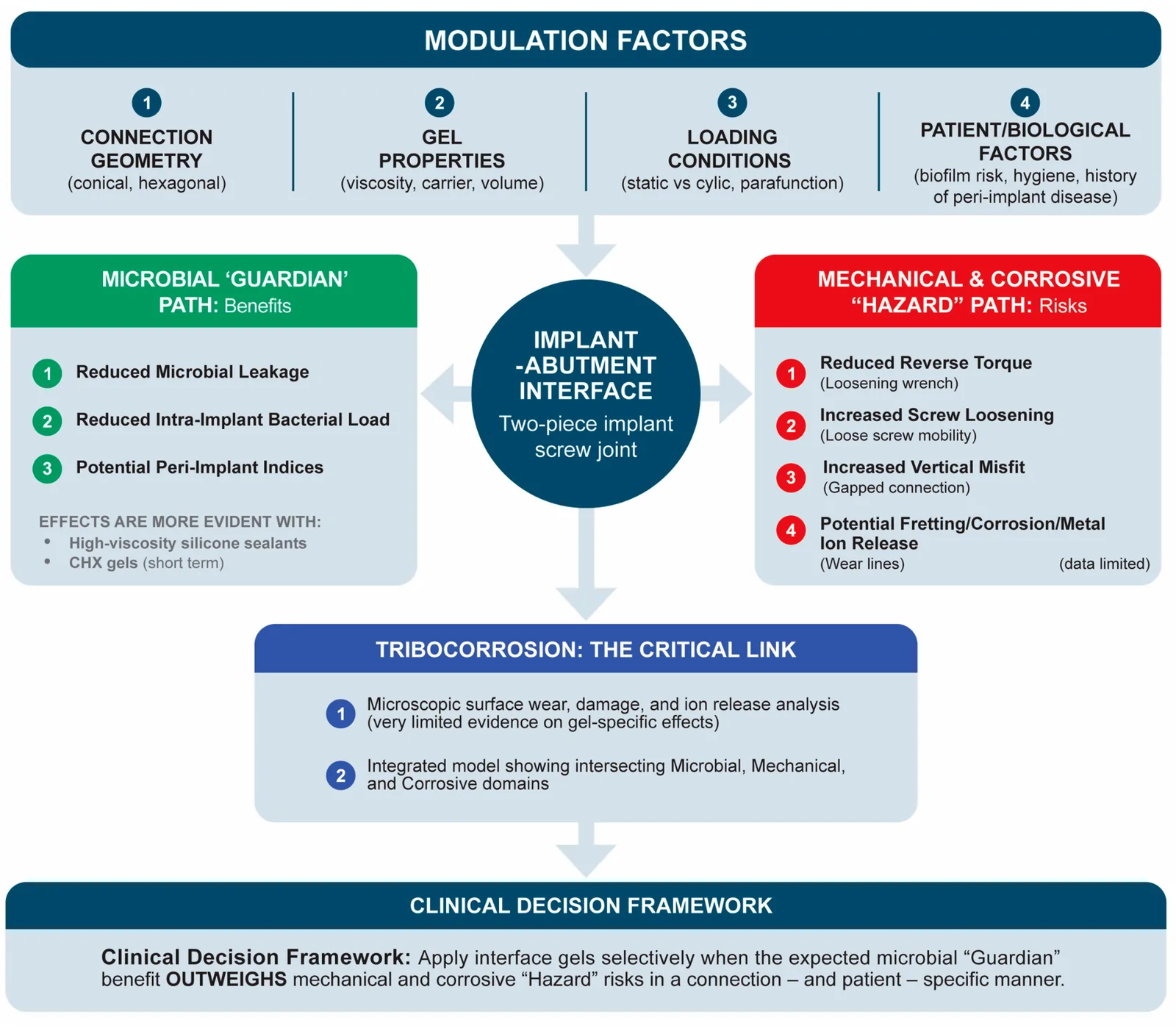
Conceptual model of implant–abutment interface gels. Antiseptic and sealing gels at the implant–abutment interface may confer microbial “Guardian” benefits by reducing leakage and intra-implant contamination but can also introduce mechanical and corrosive “Hazard” risks, particularly in specific connection geometries or with low-viscosity formulations. Connection design, gel properties, loading conditions and patient-related factors modulate the balance between these opposing effects, while tribocorrosion phenomena remain a largely unexplored but potentially critical component of the overall risk–benefit profile.

From a theoretical perspective, acidic antiseptic gels could attack the titanium oxide passivation layer, accelerating ion release. Conversely, the hermetic seal provided by silicone agents could isolate the metal surfaces from the corrosive oral electrolyte, potentially mitigating tribocorrosion. However, in the absence of direct experimental data in the included studies, this remains a theoretical hypothesis that warrants urgent future research. This conceptual positioning of tribocorrosion at the intersection of biological, mechanical and electrochemical factors is illustrated in Figure 2.

### 4.4. Clinical Implications

Based on the synthesised evidence, the use of implant–abutment interface gels should be considered a selective, indication-driven intervention rather than a routine step in every prosthetic workflow. When microbial leakage control is the principal concern, predominantly in vitro comparative studies indicate that high-viscosity silicone-based sealants may provide a more durable physical barrier than CHX-based preparations [16,48,51,53]. In several internal connection systems, these materials also maintained reverse torque values more consistently than fluid antiseptic agents [45,53]. However, this interpretation remains provisional and connection- and formulation-dependent because direct comparative clinical evidence is limited.

In contrast, the indiscriminate application of low-viscosity aqueous or oil-based CHX preparations directly within the screw channel or at the implant–abutment interface should be approached with caution. The reviewed mechanical studies consistently indicate that these agents can reduce reverse torque values and thereby increase the risk of screw loosening, particularly under cyclic loading. When CHX gels are used, they should be applied in minimal amounts and preferably limited to decontamination of the internal implant cavity rather than acting as bulk fillers of the joint. 

Implant connection geometry and prosthetic design further influence the risk–benefit profile. In steep conical connections that rely heavily on intimate metal–metal contact for frictional stability, even a thin layer of high-viscosity silicone may act as a hydraulic wedge if overdosed, preventing full seating of the abutment and increasing vertical misfit. In such designs, the threshold between beneficial sealing and mechanical interference is likely to be narrow, and the use of gels should either be avoided or restricted to very small, standardised volumes. Flat-to-flat or less tapered internal connections, on the other hand, may tolerate silicone-based sealants more safely and even benefit from their potential damping effect on micromovements.

Ultimately, the clinical decision to use an interface gel should integrate patient-related factors (biofilm risk, systemic status), implant–abutment design, prosthetic risk of loosening (e.g., cantilevers, parafunction) and the specific rheological properties of the chosen product. Rather than adopting a universal protocol, clinicians are advised to individualise gel application to cases in which the anticipated microbial “Guardian” benefit clearly outweighs the potential mechanical “Hazard”.

### 4.5. Research Gaps and Future Directions

The present scoping review highlights several important gaps in the current evidence base regarding implant–abutment interface gels. First, the predominance of in vitro studies, often conducted under highly standardised but simplified conditions, limits the extent to which their findings can be extrapolated to the complex clinical environment. Very few clinical or in vivo investigations have systematically evaluated interface gels with long-term follow-up, standardised prosthetic protocols and clearly defined peri-implant outcome measures. Well-designed prospective clinical trials, ideally incorporating both microbiological and mechanical endpoints, remain necessary.

Second, there is substantial methodological heterogeneity across existing studies. Implant systems, connection designs, torque application protocols, loading regimens, contamination models and outcome measures vary widely, hampering direct comparison and precluding quantitative synthesis. Future studies should prioritise the use of standardised protocols that more closely mimic contemporary prosthetic workflows, including defined tightening sequences, cyclic loading under biofilm challenge and clinically relevant timelines for retightening and follow-up. The consistent reporting of key variables—such as implant–abutment design, gel formulation and viscosity, torque values, loading cycles and environmental conditions—would greatly enhance comparability and facilitate more robust evidence synthesis.

Third, although CHX and silicone-based sealants have been the main focus of the literature, the range of commercially available products continues to expand, often with limited independent validation. Systematic, head-to-head comparisons of well-characterised commercial formulations are lacking, particularly for newer gels marketed with antimicrobial or sealing claims. Experimental work should move beyond single-product evaluations and adopt comparative designs that explicitly test different materials under identical conditions, thereby clarifying whether observed effects are truly material-specific or protocol-dependent.

Perhaps the most critical gap relates to corrosion and tribocorrosion phenomena at the implant–abutment interface. Only a minority of studies have explored electrochemical behaviour, metal ion release or surface degradation in the presence of interface gels, and even fewer have integrated these assessments with simultaneous microbial and mechanical testing. Future research should therefore employ integrated experimental models that concurrently evaluate microbial leakage, joint stability and electrochemical responses under dynamic loading, ideally using state-of-the-art analytical techniques for surface and ion analysis. Translating such multimodal findings into clinical studies, for example, by correlating interface gel use with peri-implant crestal bone changes, mechanical complications and biomarkers of metal exposure, would provide a more comprehensive understanding of the true Guardian and Hazard roles of these agents in everyday practice.

The present review has several limitations. The protocol was not prospectively registered, and study screening was conducted by a single reviewer, although uncertain eligibility decisions were discussed among the authors. The evidence search was based on PubMed/MEDLINE and Scopus, supplemented by ScienceDirect and reference-list screening. Although this approach was designed to cover both biomedical and materials-science literature, the omission of other bibliographic databases may have resulted in relevant studies being missed. Furthermore, the predominance of in vitro evidence and the heterogeneity of implant systems, connection designs and testing protocols limit the clinical generalisability of the findings.

## 5. Conclusions

Within the limitations of this scoping review, several consistent patterns emerge regarding the use of gels at the implant–abutment interface. CHX-based preparations appear to function primarily as short-term microbial “Guardians”, reducing intra-implant bacterial contamination and, in some clinical contexts, improving surrogate markers of peri-implant health. However, their physical sealing capability is limited and time-dependent, and their influence on joint stability is inconsistent, particularly when low-viscosity, aqueous or oil-based carriers are employed.

High-viscosity silicone-based sealants, by contrast, tend to provide more reliable microleakage control and, in several in vitro investigations, preserve or even enhance reverse torque values compared with dry controls. This suggests a potentially more favourable mechanical profile, although the predominance of in vitro data and the heterogeneity of implant systems and testing protocols warrant cautious interpretation.

Overall, implant–abutment interface gels cannot currently be recommended as a universal, one-size-fits-all solution. Their use should instead be guided by a balanced appreciation of potential Guardian benefits and Hazard risks, taking into account patient-related factors, implant–abutment design, prosthetic risk of loosening and the specific properties of each formulation. As further evidence becomes available, particularly regarding corrosion and tribocorrosion behaviour under clinically relevant conditions, these materials may be integrated into more targeted and evidence-based clinical protocols.

## Figures and Tables

**Table 1:** PCC framework for the scoping review on implant–abutment interface gels.

PCC Element	Definition in This Review	Specific Focus/Outcomes	Guiding Scoping Question(s)
Population	Human patients restored with two-piece dental implants and implant-supported restorations, as well as in vitro or in vivo experimental models replicating the implant–abutment interface	Implants with screw-retained abutments (internal or external connections), evaluated under static or dynamic loading, with or without microbial challenge	Common to all questions: “In two-piece implant systems (clinical or experimental), how do interface gels or antiseptic agents influence biological, mechanical and corrosion-related behaviour at the implant–abutment interface?”
Concept	Application of antiseptic or sealing agents (e.g., chlorhexidine gels, silicone-based sealants or similar antimicrobial interface gels) at the implant–abutment interface	Biological domain (Guardian): microbial leakage, intra-implant contamination, peri-implant inflammation indices. Mechanical domain (Hazard): reverse torque values, screw loosening, joint stability under loading. Corrosion/tribocorrosion domain: fretting corrosion, electrochemical behaviour, titanium/metal ion release or surface degradation in the presence of gels.	Q1—Microbial leakage (Guardian):“Do antiseptic gels effectively prevent or reduce microbial leakage at the implant–abutment interface?” Q2—Mechanical stability (Hazard):“Does the application of these agents compromise or enhance the mechanical stability of the implant–abutment assembly (e.g., reverse torque, screw loosening)?” Q3—Corrosion/tribocorrosion (Hazard):“Does the use of antiseptic gels or related sealing agents promote, mitigate, or otherwise influence corrosion and tribocorrosion-related phenomena (e.g., fretting corrosion, titanium release) at the implant–abutment interface?”
Context	Clinical and experimental settings involving two-piece endosseous implant systems where the implant–abutment interface is a primary variable of interest	Screw-retained implant restorations with a defined implant–abutment interface (internal or external), assessed under conditions simulating the oral environment (thermal cycling, mechanical loading, or microbial challenge), in which interface gels are compared with dry or contaminated controls	“In the context of two-piece implant systems and screw-retained restorations, what is the overall balance between the ‘Guardian’ (biological) benefits and the ‘Hazard’ (mechanical and corrosion-related) risks associated with the use of interface gels at the implant–abutment connection?”

**Table 3:** Overview of commercially available implant–abutment interface sealing agents. Products are listed in alphabetical order.

Product Name	Manufacturer	Composition	Viscosity/Nature
AbutmentFix^®^	Novis Medical (Ra’anana, Israel)	low viscosity addition curing vinyl silicone	low viscosity
flow.sil	Bredent medical GmbH & Co. KG (Senden, Germany)	poly-dimethyl-siloxane-based	Thin, flowable silicone gel that hardens 4 min. after placement
GapSeal^®^	Hager & Werken (Duisburg, Germany)	Silicone matrix ingredient composition	High-viscosity paste
ImplaSeal^®^	Dentakote (Boca Raton, FL, USA)	Proprietary silicone-based polymer	Flowable paste
IMPLAseal NEXT	NEXT (Tel Aviv, Israel)	Silicone matrix gel with thymol	Not disclosed
Kiero Seal^®^ Gel	Kuss Dental (Madrid, Spain)	Polyvinylsiloxane (siloxane)-based gel; Shore A hardness ≈ 18	Thixotropic polyvinylsiloxane gel; medium–high viscosity (≈350 Pa)
OxySafe^®^ Gel/OxySafe Gel Professional	Hager & Werken (Germany)	Active-oxygen gel based on Ardox-X (hydrocarbon-oxo-borate complex); CHX/peroxide-free	Low- to medium-viscosity oxygen-releasing gel
Sil-Flow^®^	DSI Ltd. (Ashdod, Israel)	Silicone matrix with light-curing functionality	Viscous light-curing flowable silicone-resin gel
ViruSeal^®^	DSI Ltd. (Israel)	Silicone gel polymer	Viscous gel/paste

Data sources: Manufacturer instructions for use (IFU) and/or safety data sheets (SDS) and official product pages (see Supplementary Table S3); accessed 13 December 2025.

## Data Availability

All data charted and analysed in this scoping review are included in this article and its Supplementary Materials.

## Supplementary Materials

The following supporting information can be downloaded at https://www.mdpi.com/article/10.3390/dj14100671/s1.

## Abbreviations

The following abbreviations are used in this manuscript:

CHX	chlorhexidine
IAI	implant–abutment interface
IFU	instructions for use
PCC	Population–Concept–Context
PRISMA-ScR	Preferred Reporting Items for Systematic Reviews and Meta-Analyses extension for Scoping Reviews
RTV	reverse torque value(s)
SDS	safety data sheet(s)

## References

[1] Pjetursson B.E., Thoma D., Jung R., Zwahlen M., Zembic A. (2012). A systematic review of the survival and complication rates of implant-supported fixed dental prostheses (FDP s) after a mean observation period of at least 5 years. Clin. Oral Implant. Res..

[2] Berglundh T., Persson L., Klinge B. (2002). A systematic review of the incidence of biological and technical complications in implant dentistry reported in prospective longitudinal studies of at least 5 years. J. Clin. Periodontol..

[3] Jung R.E., Pjetursson B.E., Glauser R., Zembic A., Zwahlen M., Lang N.P. (2008). A systematic review of the 5-year survival and complication rates of implant-supported single crowns. Clin. Oral Implant. Res..

[4] Mao Z., Beuer F., Wu D., Zhu Q., Yassine J., Schwitalla A., Schmidt F. (2023). Microleakage along the implant–abutment interface: A systematic review and meta-analysis of in vitro studies. Int. J. Implant Dent..

[5] Kowalski J., Puszkarz A.K., Radwanski M., Sokolowski J., Cichomski M., Bourgi R., Hardan L., Sauro S., Lukomska-Szymanska M. (2023). Micro-CT Evaluation of Microgaps at Implant–Abutment Connection. Materials.

[6] D’Ercole S., Dotta T.C., Iezzi G., Cipollina A., Pedrazzi V., Piattelli A., Petrini M. (2023). Static bacterial leakage in different conometric connections: An in vitro study. Appl. Sci..

[7] Romanos G.E., Malmstrom H., Feng C., Ercoli C., Caton J. (2014). Immediately loaded platform-switched implants in the anterior mandible with fixed prostheses: A randomized, split-mouth, masked prospective trial. Clin. Implant Dent. Relat. Res..

[8] Tesmer M., Wallet S., Koutouzis T., Lundgren T. (2009). Bacterial colonization of the dental implant fixture–abutment interface: An in vitro study. J. Periodontol..

[9] Kowalski J., Lapinska B., Nissan J., Lukomska-Szymanska M. (2021). Factors influencing marginal bone loss around dental implants: A narrative review. Coatings.

[10] Romanos G.E., Biltucci M.T., Kokaras A., Paster B.J. (2016). Bacterial composition at the implant-abutment connection under loading in vivo. Clin. Implant Dent. Relat. Res..

[11] Ghannad F., Alkadi L.T., Wiebe C.B., Shen Y., Haapasalo M., Larjava H.S. (2015). Intra-operative application of chlorhexidine gel reduces bacterial counts in internal implant cavity. Eur. J. Oral Sci..

[12] Ali A.K., Basima G., Abbas S. (2017). Potential effect of 2% chlorhexidine gel in the implant screw hole on bacterial count. Int. J. Med. Res. Health Sci..

[13] Asbi T., Hussein H.A., Horwitz J., Gabay E., Machtei E.E., Giladi H.Z. (2021). A single application of chlorhexidine gel reduces gingival inflammation and interleukin 1-β following one-stage implant placement: A randomized controlled study. Clin. Implant Dent. Relat. Res..

[14] Mathur S., Mathur T., Srivastava R., Khatri R. (2011). Chlorhexidine: The gold standard in chemical plaque control. Natl. J. Physiol. Pharm. Pharmacol..

[15] Abraham H.M., Philip J.M., Kruppa J., Jain A.R., Krishnan C.V. (2015). Use of chlorhexidine in implant dentistry. Biomed. Pharmacol. J..

[16] Raju S., Harshakumar K., Ravichandran R., Nair V.V., Janardanan K., Francis L. (2021). Evaluation of the Sealing Ability of Antibacterial Sealing Gel Versus Chlorhexidine Varnish at the Implant-Abutment Interface: An: In vitro Study. J. Interdiscip. Dent..

[17] Mencio F., Papi P., Di Carlo S., Pompa G. (2016). Salivary bacterial leakage into implant-abutment connections: Preliminary results of an in vitro study. Eur. Rev. Med. Pharmacol. Sci..

[18] Collins J.R., Veras K., Hernández M., Hou W., Hong H., Romanos G.E. (2021). Anti-inflammatory effect of salt water and chlorhexidine 0.12% mouthrinse after periodontal surgery: A randomized prospective clinical study. Clin. Oral Investig..

[19] Kern M., Harder S. (2010). Antimicrobial filling of implant cavities. J. Prosthet. Dent..

[20] Sagheb K., Görgen C.-I., Döll S., Schmidtmann I., Wentaschek S. (2023). Preload and friction in an implant–abutment–screw complex including a carbon-coated titanium alloy abutment screw: An in vitro study. Int. J. Implant Dent..

[21] Wentaschek S., Tomalla S., Schmidtmann I., Lehmann K.M. (2017). Preload, Coefficient of Friction, and Thread Friction in an Implant-Abutment-Screw Complex. Int. J. Prosthodont..

[22] Jörn D., Kohorst P., Besdo S., Rücker M., Stiesch M., Borchers L. (2014). Influence of lubricant on screw preload and stresses in a finite element model for a dental implant. J. Prosthet. Dent..

[23] Rathe F., Ratka C., Kaesmacher C., Winter A., Brandt S., Zipprich H. (2021). Influence of different agents on the preload force of implant abutment screws. J. Prosthet. Dent..

[24] Sun M., Shui Y., Zhang Y., Ma R., Zhao Y., Chen H., Yu P., Li Z., Wu T., Yu H. (2024). Influence of Abutment Screw-Tightening Methods on the Screw Joint: Immediate and Long-Term Stability. Int. J. Dent..

[25] Wu T., Fan H., Ma R., Chen H., Li Z., Yu H. (2017). Effect of lubricant on the reliability of dental implant abutment screw joint: An in vitro laboratory and three-dimension finite element analysis. Mater. Sci. Eng. C.

[26] Bulaqi H.A., Mashhadi M.M., Geramipanah F., Safari H., Paknejad M. (2015). Effect of the coefficient of friction and tightening speed on the preload induced at the dental implant complex with the finite element method. J. Prosthet. Dent..

[27] Yang T.-H., Chuang K.-J., Masumi S.-I., Lin H.-W. (2016). Comparison of preload values of dental implant abutment screws subjected to surface and non-surface treatments. J. Kyushu Dent. Soc..

[28] Colpak E.D., Gumus H.O. (2020). Effect of surface modifications of abutment screws on reverse torque values: An in vitro study. Int. J. Prosthodont..

[29] Jung R.E., Zembic A., Pjetursson B.E., Zwahlen M., Thoma D.S. (2012). Systematic review of the survival rate and the incidence of biological, technical, and aesthetic complications of single crowns on implants reported in longitudinal studies with a mean follow-up of 5 years. Clin. Oral Implant. Res..

[30] Bishti S., Alsagher M.S.E., Homa M., Wolfart S., Tuna T. (2025). Factors Influencing the Screw Stability of Implant-Supported Single Crowns: An In Vitro Study. Materials.

[31] Theoharidou A., Petridis H.P., Tzannas K., Garefis P. (2008). Abutment screw loosening in single-implant restorations: A systematic review. Int. J. Oral Maxillofac. Implant..

[32] Dini C., Costa R.C., Sukotjo C., Takoudis C.G., Mathew M.T., Barão V.A. (2020). Progression of bio-tribocorrosion in implant dentistry. Front. Mech. Eng..

[33] Alhamad M., Barão V.A., Sukotjo C., Cooper L.F., Mathew M.T. (2021). Ti-ions and/or particles in saliva potentially aggravate dental implant corrosion. Materials.

[34] Nourizadeh A., Shafiee E., Khorramdel A., Mousavi S.A., Rahbar M. (2022). Comparison of reverse torque values of abutment screws with the application of oil-based and water-based antibacterial agents. J. Dent. Res. Dent. Clin. Dent. Prospect..

[35] Jervøe-Storm P.M., Jepsen S., Marder M., Kraus D., Stoilov M., Enkling N. (2023). Prevention of internal bacterial colonization of dental implants: A comparative longitudinal observational study. Clin. Oral Implant. Res..

[36] Mostafavi A.S., Memarian M., Seddigh M.A. (2021). Effect of fluid contamination on reverse torque values in implant-abutment connections under oral conditions. J. Adv. Prosthodont..

[37] Sun F., Cheng W., Zhao B.-H., Song G.-Q., Lin Z. (2022). Evaluation the loosening of abutment screws in fluid contamination: An in vitro study. Sci. Rep..

[38] Asli H.N., Saberi B.V., Fatemi A.S. (2017). In vitro effect of chlorhexidine gel on torque and detorque values of implant abutment screw. Indian J. Dent. Res..

[39] Sinjari B., D’Addazio G., De Tullio I., Traini T., Caputi S. (2018). Peri-implant bone resorption during healing abutment placement: The effect of a 0.20% chlorhexidine gel vs. placebo—A randomized double blind controlled human study. BioMed Res. Int..

[40] D’Addazio G., Manciocchi E., Tafuri G., Schiavone R., Murmura G., Mavriqi L., Sinjari B., Caputi S. (2023). Long-Term Efficacy of Chlorhexidine Gel in Single-Crown Implant Rehabilitation: A Five-Year Follow-Up Study. Dent. J..

[41] Fiorillo L. (2019). Chlorhexidine gel use in the oral district: A systematic review. Gels.

[42] Tricco A.C., Lillie E., Zarin W., O’Brien K.K., Colquhoun H., Levac D., Moher D., Peters M.D., Horsley T., Weeks L. (2018). PRISMA extension for scoping reviews (PRISMA-ScR): Checklist and explanation. Ann. Intern. Med..

[43] Page M.J., McKenzie J.E., Bossuyt P.M., Boutron I., Hoffmann T.C., Mulrow C.D., Shamseer L., Tetzlaff J.M., Akl E.A., Brennan S.E. (2021). The PRISMA 2020 statement: An updated guideline for reporting systematic reviews. BMJ.

[44] Podhorsky A., Biscoping S., Rehmann P., Streckbein P., Domann E., Woestmann B. (2016). Transfer of bacteria into the internal cavity of dental implants after application of disinfectant or sealant agents in vitro. Int. J. Oral Maxillofac. Implant..

[45] Katebi T., Heidari S., Aalaei S. (2024). Effect of a silicon-based sealing agent (GapSeal) on the reverse torque value of the abutment-implant screw. J. Dent. Mater. Tech..

[46] Ozdiler A., Dayan S.C., Gencel B., Ozkol G.I. (2021). Reverse torque values of abutment screws after dynamic loading: Effects of sealant agents and the taper of conical connections. J. Oral Implantol..

[47] Koutouzis T., Gadalla H., Kettler Z., Elbarasi A., Nonhoff J. (2015). The role of chlorhexidine on endotoxin penetration to the implant-abutment interface (IAI). Clin. Implant Dent. Relat. Res..

[48] Smojver I., Bjelica R., Vuletić M., Gerbl D., Budimir A., Gabrić D. (2022). Antimicrobial Efficacy and Permeability of Various Sealing Materials in Two Different Types of Implant–Abutment Connections. Int. J. Mol. Sci..

[49] Antonio Ferreira L., Eduardo Francischone C., Salles Sotto-Maior B., Pedroso Turssi C., Edson Miranda M., de Vasconcellos A.A., de Carvalho R.S. (2017). Effectiveness of Kiero seal material against the bacterial infiltration on the implant/abutment interface. Int. J. Clin. Dent..

[50] Cao X., Cai X., Chen R., Zhang H., Jiang T., Wang Y. (2019). A thermosensitive chitosan-based hydrogel for sealing and lubricating purposes in dental implant system. Clin. Implant Dent. Relat. Res..

[51] Mohammadi F., Hajmousaei M., Vaziri N., Arshad M. (2019). Bacterial leakage at implant-abutment interface with different intermediate materials. J. Oral Implantol..

[52] Seloto C.B., Strazzi-Sahyon H.B., Dos Santos P.H., Assunção W.G. (2020). Effectiveness of Sealing Gel on Vertical Misfit at the Implant-Abutment Interface and Preload Maintenance of Screw-Retained Implant-Supported Prostheses. Int. J. Oral Maxillofac. Implant..

[53] Yu P., Li Z., Tan X., Yu H. (2022). Effect of sealing gel on the microleakage resistance and mechanical behavior during dynamic loading of 3 implant systems. J. Prosthet. Dent..

