# Supplementary material for: Implant–Abutment Interface Gels in Two-Piece Implant Systems: The “Guardian–Hazard” Paradigm—A Scoping Review
Source: Dent J (Basel). 2026 Oct 14;14(10):671. doi: 10.3390/dj14100671 (PMC13703747; doi:10.3390/dj14100671)
Supplement: Supplementary file 1 [file dentistry-14-00671-s001.zip › Supplementary Material S1_tables.pdf]

Supplementary Table S1. Full electronic search strategies and yields (executed on 13 & 14 Dec 2025).

**Research Database/ Platform**	**Date searched**	**Fields searched**	**Limits/filters**	**Search string**	**Records retrieved**
PubMed/ MEDLINE	14 Dec 2025	MeSH + Title/Abstract (tiab)	Publication date: 2015/01/01–2025/12/12;	a) Microbial Leakage<br>(("Dental Implants"[Mesh] OR dental implant*[tiab] OR oral implant*[tiab]) AND (abutment*[tiab] OR "Dental Abutments"[Mesh] OR "implant abutment"[tiab] OR "implant-abutment"[tiab] OR "implant-abutment interface"[tiab] OR "implant-abutment junction"[tiab] OR "implant-abutment connection"[tiab] OR IAJ[tiab] OR "morse taper"[tiab] OR conical[tiab]) AND (chlorhexidine[tiab] OR CHX[tiab] OR "chlorhexidine digluconate"[tiab] OR ("chlorhexidine"[tiab] AND gel[tiab]) OR "chlorhexidine gel"[tiab] OR silicone[tiab] OR polydimethylsiloxane[tiab] OR dimethicone[tiab] OR "silicone sealant"[tiab] OR sealant*[tiab] OR sealing[tiab] OR(gel[tiab] OR gels[tiab] OR "gel-based"[tiab])) AND (microbial[tiab] OR bacteria*[tiab] OR biofilm[tiab] OR endotoxin*[tiab] OR "bacterial leakage"[tiab] OR "microbial leakage"[tiab] OR microleakage[tiab] OR contamination[tiab] OR CFU[tiab]))<br>b) Mechanical stability<br>(("Dental Implants"[Mesh] OR dental implant*[tiab] OR oral implant*[tiab]) AND (abutment*[tiab] OR "Dental Abutments"[Mesh] OR "implant abutment"[tiab] OR "implant-abutment"[tiab] OR "implant-abutment interface"[tiab] OR "implant-abutment junction"[tiab] OR "implant-abutment connection"[tiab] OR IAJ[tiab] OR "morse taper"[tiab] OR conical[tiab]) AND (chlorhexidine[tiab] OR CHX[tiab] OR "chlorhexidine digluconate"[tiab] OR ("chlorhexidine"[tiab] AND gel[tiab]) OR "chlorhexidine gel"[tiab] OR silicone[tiab] OR polydimethylsiloxane[tiab] OR dimethicone[tiab] OR "silicone sealant"[tiab] OR sealant*[tiab] OR sealing[tiab] OR "sealing gel"[tiab]) AND ("reverse torque"[tiab] OR detorque*[tiab] OR "removal torque"[tiab] OR preload[tiab] OR "screw loosening"[tiab] OR "torque loss"[tiab] OR "torque maintenance"[tiab] OR settling[tiab] OR "embedment relaxation"[tiab] OR micromovement[tiab] OR "cyclic loading"[tiab] OR fatigue[tiab] OR friction[tiab] OR "coefficient of friction"[tiab]))<br>c) Corrosion/ tribocorrosion<br>(("Dental Implants"[Mesh] OR dental implant*[tiab] OR oral implant*[tiab]) AND (abutment*[tiab] OR "Dental Abutments"[Mesh] OR "implant abutment"[tiab] OR "implant-abutment"[tiab] OR "implant-abutment interface"[tiab] OR "implant-abutment junction"[tiab] OR "implant-abutment connection"[tiab] OR IAJ[tiab] OR "morse taper"[tiab] OR conical[tiab]) AND (chlorhexidine[tiab] OR CHX[tiab] OR "chlorhexidine digluconate"[tiab] OR ("chlorhexidine"[tiab] AND gel[tiab]) OR "chlorhexidine gel"[tiab] OR silicone[tiab] OR polydimethylsiloxane[tiab] OR dimethicone[tiab] OR "silicone sealant"[tiab] OR sealant*[tiab] OR sealing[tiab] OR "sealing gel"[tiab]) AND (corrosion[tiab] OR tribocorrosion[tiab] OR fretting[tiab] OR "fretting corrosion"[tiab] OR galvanic[tiab] OR electrochemical[tiab] OR "ion release"[tiab] OR "metal release"[tiab] OR "open circuit potential"[tiab] OR potentiodynamic[tiab] OR EIS[tiab] OR pitting[tiab] OR "crevice corrosion"[tiab] OR "wear debris"[tiab]))	Microbial leakage<br>n = 87;<br>Mechanical stability<br>n = 24;<br>Corrosion/ tribocorrosion n = 3;<br>Total n = 114

Scopus	14 Dec 2025	TITLE-ABS-KEY	PUBYEAR 2015–2025; DOCTYPE=article; LANGUAGE=English; SUBJAREA=Dentistry applied to microbial and mechanical searches	a) Microbial Leakage<br>TITLE-ABS-KEY ("dental implant" OR "dental implants" OR "oral implant" OR "oral implants")<br>AND TITLE-ABS-KEY (abutment OR abutments OR "implant abutment" OR "implant-abutment" OR "implant-abutment interface" OR "implant-abutment junction" OR "implant-abutment connection" OR IAJ OR "morse taper" OR conical)<br>AND TITLE-ABS-KEY (chlorhexidine OR CHX OR "chlorhexidine digluconate" OR "chlorhexidine gel" OR silicone OR polydimethylsiloxane OR dimethicone OR "silicone sealant" OR sealant OR sealing OR "sealing gel")<br>AND TITLE-ABS-KEY (microbial OR bacterial OR bacteria OR biofilm OR endotoxin OR microleakage OR contamination OR CFU OR "bacterial leakage" OR "microbial leakage")<br>b) Mechanical stability<br>TITLE-ABS-KEY ("dental implant" OR "dental implants" OR "oral implant" OR "oral implants") AND TITLE-ABS-KEY (abutment OR abutments OR "implant abutment" OR "implant-abutment" OR "implant-abutment interface" OR "implant-abutment junction" OR "implant-abutment connection" OR IAJ OR "morse taper" OR conical) AND TITLE-ABS-KEY (chlorhexidine OR CHX OR "chlorhexidine digluconate" OR "chlorhexidine gel" OR silicone OR polydimethylsiloxane OR dimethicone OR "silicone sealant" OR sealant OR sealing OR "sealing gel") AND TITLE-ABS-KEY ("reverse torque" OR detorque OR "removal torque" OR preload OR "screw loosening" OR "torque loss" OR "torque maintenance" OR settling OR micromovement OR "cyclic loading" OR fatigue OR friction) AND (LIMIT-TO (SUBJAREA , "DENT")) AND (LIMIT-TO (DOCTYPE , "ar"))<br>c) Corrosion/ tribocorrosion<br>TITLE-ABS-KEY ("dental implant" OR "dental implants" OR "oral implant" OR "oral implants") AND TITLE-ABS-KEY (abutment OR abutments OR "implant abutment" OR "implant-abutment" OR "implant-abutment interface" OR "implant-abutment junction" OR "implant-abutment connection" OR IAJ OR "morse taper" OR conical) AND TITLE-ABS-KEY (chlorhexidine OR CHX OR "chlorhexidine digluconate" OR "chlorhexidine gel" OR silicone OR polydimethylsiloxane OR dimethicone OR "silicone sealant" OR sealant OR sealing OR "sealing gel") AND TITLE-ABS-KEY (corrosion OR tribocorrosion OR fretting OR galvanic OR electrochemical OR "ion release" OR "metal release" OR "open circuit potential" OR potentiodynamic OR EIS OR pitting OR "crevice corrosion") AND PUBYEAR > 2014 AND PUBYEAR < 2026 AND (LIMIT-TO (DOCTYPE , "ar"))	Microbial leakage n = 61; Mechanical stability n = 26; Corrosion/tribocorrosion n = 5; Total n = 92
ScienceDirect	13 Dec 2025	Advanced search (platform default fields)	Years 2015–2025; Research articles	("implant abutment" OR "implant-abutment") AND (microgap OR microleak) AND (chlorhexidine OR CHX OR seal)	n = 35

Supplementary Table S2. Decision rules for Guardian/Hazard coding (operationalised).		
Outcome domain	“Guardian” (benefit vs control)	“Hazard” (risk vs control)
Microbial sealing/contamination	↓ microleakage / ↓ CFU / ↓ endotoxin ingress	↑ leakage / ↑ CFU / ↑ ingress
Mechanical stability	↑ RTV / ↓ torque loss / ↓ screw loosening	↓ RTV / ↑ torque loss / ↑ loosening
Electrochemical/corrosion	↓ corrosion current/ion release/fretting indicators	↑ corrosion/ion release/fretting indicators
Multi-domain studies	If directions agree → single label	If discordant → **Mixed (Guardian + Hazard)**
Operational definition (Guardian/Hazard): For each included study, we coded outcomes by domain (microbial sealing/contamination, mechanical stability, electrochemical/corrosion). A result was coded as “Guardian” when the tested agent produced a beneficial direction of effect versus the comparator within the stated domain (e.g., reduced leakage/CFU/endotoxin ingress; increased reverse torque values or reduced screw loosening; reduced corrosion/ion release). A result was coded as “Hazard” when the agent produced a detrimental direction of effect versus the comparator (e.g., increased leakage/contamination; reduced reverse torque or increased loosening; increased corrosion markers). When a study reported multiple domains and the directionality was discordant, we coded it as “Mixed (Guardian + Hazard)” and reported domain-specific labels rather than forcing a single global classification.		

Supplementary Table S3. Source documents for the commercial sealing agents listed in main-text Table 3.

Product	Brand	Source document(s)	URL(s)
AbutmentFix	Novis Medical (Israel)	Product page	https://www.norismedical.com/abutmentfix
flow.sil	bredent medical (Germany)	Product page; Product brochure (PDF)	https://www.bredent-implants.com/products-solutions/tools/flow-sil/<br>https://bredent-group.com/wp-content/uploads/2019/12/flow-sil_000533GB-20150220_flow-sil_210x297mm.pdf
GapSeal	Hager & Werken (Germany)	Product page; IFU (PDF); Handling/Reprocessing (PDF)	https://www.hagerwerken.de/en/katalog/dentist/gapseal-en/<br>https://www.hagerwerken.de/wp-content/uploads/2018/09/GapSeal-GBA-16S-A7-Rev05-0918-kl-1.pdf<br>https://www.hagerwerken.de/wp-content/uploads/2017/05/GapSeal-WBA-0317.pdf
ImplaSeal	DentaKote / Dentity Global (USA)	Product page; Resources hub; SDS (PDF); IFU (PDF)	https://dentakote.com/implaseal/<br>https://dentakote.com/resources/<br>https://dentakote.com/wp-content/uploads/2022/09/SDS-Sheet-ImplaSeal-Final.pdf<br>https://dentakote.com/wp-content/uploads/2022/10/ImplaSeal-Instructions-for-use.pdf
IMPLAseal NEXT	*(Distributor listing)*	Distributor listing page	https://tz-trade.com/dental-implants/surgical-materials/implaseal-gaps-sealant-for-dental-implants
OxySafe Gel Professional	Hager & Werken (Germany)	Product page; IFU Gel (PDF); IFU Liquid (PDF); (Optional) Catalogue PDF	https://www.hagerwerken.de/en/katalog/dentist/miradent-en/oxysafe-gel-professional-en/<br>https://www.hagerwerken.de/wp-content/uploads/2021/02/OXYSAFE-Gel-GA-0420-Homepage-kl.pdf<br>https://www.hagerwerken.de/wp-content/uploads/2021/02/OXYSAFE-Liquid-GBA-0420Homepage-kl.pdf<br>https://www.hagerwerken.de/wp-content/uploads/2023/03/HW-Praxiskatalog-20-GB-Rev1-0223-web.pdf
Sil-Flow	DSI (Israel)	Official product page (resources for IFU/MSDS)	https://dsisrael.com/product/sil-flow/
ViruSeal	DSI (Israel)	Official product page (resources for IFU/MSDS)	https://www.dsisrael.com/product-search?*=ViruSeal+Gel<br>https://cdn.prod.website-files.com/62824b14dce5c0a803fd462d/63301c0458d0cafb5525f05f_631b462a0b99804b18112e0f_ViruSeal-SilFlow_A4_EN_print_Page_1.jpg<br>https://cdn.prod.website-files.com/62824b13dce5c09ad3fd4623/631b49a226aca4157b8858da_DSI_ViruSeal_Ifu.pdf<br>https://cdn.prod.website-files.com/62824b13dce5c09ad3fd4623/631b4603a2ff1c5268ea05e4_DSI_MSDS-ViruSeal.pdf
Kiero Seal Gel	Kuss Dental (Spain)	Product page; IFU (PDF); Brochure (PDF)	https://kuss-dental.com/wp-content/uploads/2023/11/folleto_Kiero_Seal.pdf<br>https://kuss-dental.com/wp-content/uploads/2023/11/Kiero_Seal_eng.pdf

All URLs were accessed on 13 December 2025
